# Short-term preliminary functional results from HIPOCRAT-ESK study: a cohort, non-interventional, prospective, real-world study of functional remission in TRD patients treated with Esketamina nasal spray.

**DOI:** 10.1192/j.eurpsy.2026.11436

**Published:** 2026-07-31

**Authors:** T. Caraballo Lopez, M. García Dorado, L. Gutiérrez - Rojas, J. M. Olivares, N. Cardoner Álvarez, Á. Ibáñez Cuadrado

**Affiliations:** 1 Neuroscience; 2 Johnson & Johnson, Madrid; 3 Hospital San Cecilio, Granada; 4 Hospital Álvaro Cunqueiro, Vigo; 5 Hospital Sant Pau i Santa Creu, Barcelona; 6 Hospital Ramón y Cajal, Madrid, Spain

## Abstract

**Introduction:**

Treatment resistant depression (TRD) is related to more severe disease burden, including poorer health, quality of life, and productivity, exacerbated by the lack of effective treatments. Delayed appropriate treatment is associated with mediocre clinical outcomes, longer time to remission, and poorer functional outcomes. Potential for functional improvement decreases with symptom chronicity and treatment failure. Esketamine nasal spray (ESK NS), with its promising results, offers potential to improve TRD patient outcomes. HOIPOCRAT-ESK is a real-world study designed to assess functional remission rates in TRD patients under ESK NS treatment at different TRD stages, evaluating if early intervention relates to more beneficial functional and clinical outcomes in Spanish clinical practice.

**Objectives:**

To report the initial functional results of HIPOCRAT-ESK, the first real-world, prospective study evaluating functional remission of TRD patients treated with ESK NS based in Spanish clinical practice.

**Methods:**

HIPOCRAT-ESK is a cohort, non-interventional, prospective, real-world study evaluating functions remission as a primary endpoint in TRD patients treated with ESK NS as per the Spanish clinical practice including a total sample of 300 TRD patients with 12 months prospective follow-up. Functional evolution diuring treatment is evaluated with SDS scale. Functional remission was defined as total SDS score of ≤6.

**Results:**

83 patients were included in the intention to treat analysis. Patient’s basal functioning was severely impacted (total SDS basal score = 23.48 points). In this hard-to-treat population, ESK NS achieved positive functional results (Figure 1), with sustained improvement in functioning during induction pahse (mean SDS decrease of 3.96 and 6.04 points by day 15 and 28 of treatment, respectively). 10% of patients achieved functional remission status by the end of the induction phase (higher funtional remission rates were obserd in early TRD patients compared to chronic TRD ones). Highlighting the key importance of early treatment optimization.

**Table 1.** SDS Total score evolution: Table 1. Long description.

	Visit 1 (Day 0)	Visit 2 (Day 15)	Visit 3 (Day 28)
**Number of participants**	35	34	29
**Mean SDS (SD)**	24.46 (5.15)	20.50 (7.71)	18.41 (8.23)
**Mean SDS decrease vs basal**		–3.96	–6.04

**Table 2.** Functional remission rates: Table 2. Long description.

POPULATION	ITT		TRD4		TRD5		TRD6+	
INDUCTION PHASE DAY	Day 15	Day 28	Day 15	Day 28	Day 15	Day 28	Day 15	Day 28
**Functional remission n (%)**	3 (3.9%)	7 (10%)	1 (2.94%)	2 (6.90%)	2 (9.52%)	4 (20%)	0,00	1 (4.76%)

**Conclusions:**

Preliminary functional short-term analysis from HIPOCRAT-ESK stuyd show promising functional improvements results, observed as soon as second administration session, and sustained over the induction phase. The positive results were more marked in early TRD profiles vs chronic TRD patients.

**Disclosure of Interest:**

T. Caraballo Lopez Employee of: Full time J&J employee, M. García Dorado Employee of: Johnson & Johnson Full-time employee, L. Gutiérrez - Rojas Grant / Research support from: Has recieved research support from Johnson & Johnson, Consultant of: Has participated as consultant for Johnson & Johnson, J. M. Olivares Grant / Research support from: Has recieved research support from Johnson & Johnson, Consultant of: Has participated as consultant for Johnson & Johnson, N. Cardoner Álvarez Grant / Research support from: Has recieved Research support from Johnson & Johnson, Consultant of: Has participated as consultant for Johnson & Johnson, Á. Ibáñez Cuadrado Grant / Research support from: Has recieved research support from Johnson & Johnson, Consultant of: Has participated as consultant fro Johnson & Johnson

## Long descriptions

### Table 1. Long description

The table consists of four columns and four rows.

Column headers from left to right are: Blank, Visit 1 (Day 0), Visit 2 (Day 15), and Visit 3 (Day 28).

Row 1: Number of participants. Visit 1 has 35; Visit 2 has 34; Visit 3 has 29.

Row 2: Mean S D S (S D). Visit 1 is 24.46 (5.15); Visit 2 is 20.50 (7.71); Visit 3 is 18.41 (8.23).

Row 3: Mean S D S decrease versus basal. Visit 1 is blank; Visit 2 shows a decrease of minus 3.96; Visit 3 shows a decrease of minus 6.04.

Navigate back to Table 1..

### Table 2. Long description

The table consists of nine columns. The first column lists the metric Functional remission n (%). The remaining columns are grouped by population: I T T, T R D 4, T R D 5, and T R D 6 plus. Each population is subdivided into two time points: Day 15 and Day 28.

* I T T: 3 (3.9%) at Day 15 and 7 (10%) at Day 28.

* T R D 4: 1 (2.94%) at Day 15 and 2 (6.90%) at Day 28.

* T R D 5: 2 (9.52%) at Day 15 and 4 (20%) at Day 28.

* T R D 6 plus: 0,00 at Day 15 and 1 (4.76%) at Day 28.

Navigate back to Table 2..

